# Age related extracellular matrix and interstitial cell phenotype in pulmonary valves

**DOI:** 10.1038/s41598-020-78507-8

**Published:** 2020-12-07

**Authors:** Shaohua Wu, Vikas Kumar, Peng Xiao, Mitchell Kuss, Jung Yul Lim, Chittibabu Guda, Jonathan Butcher, Bin Duan

**Affiliations:** 1 College of Textiles & Clothing, Qingdao University, Qingdao, People’s Republic of China; 2 Mary & Dick Holland Regenerative Medicine Program and Division of Cardiology, Department of Internal Medicine, University of Nebraska Medical Center, Omaha, NE USA; 3 Mass Spectrometry and Proteomics Core Facility, University of Nebraska Medical Center, Omaha, NE USA; 4 Department of Genetics, Cell Biology and Anatomy, College of Medicine, University of Nebraska Medical Center, Omaha, NE USA; 5 Department of Mechanical and Materials Engineering, University of Nebraska-Lincoln, Lincoln, NE USA; 6 Meinig School of Biomedical Engineering, Cornell University, Ithaca, NY USA; 7 Department of Surgery, College of Medicine, University of Nebraska Medical Center, Omaha, NE USA

**Keywords:** Biomarkers, Cardiology

## Abstract

Heart valve disease is a common manifestation of cardiovascular disease and is a significant cause of cardiovascular morbidity and mortality worldwide. The pulmonary valve (PV) is of primary concern because of its involvement in common congenital heart defects, and the PV is usually the site for prosthetic replacement following a Ross operation. Although effects of age on valve matrix components and mechanical properties for aortic and mitral valves have been studied, very little is known about the age-related alterations that occur in the PV. In this study, we isolated PV leaflets from porcine hearts in different age groups (~ 4–6 months, denoted as young versus ~ 2 years, denoted as adult) and studied the effects of age on PV leaflet thickness, extracellular matrix components, and mechanical properties. We also conducted proteomics and RNA sequencing to investigate the global changes of PV leaflets and passage zero PV interstitial cells in their protein and gene levels. We found that the size, thickness, elastic modulus, and ultimate stress in both the radial and circumferential directions and the collagen of PV leaflets increased from young to adult age, while the ultimate strain and amount of glycosaminoglycans decreased when age increased. Young and adult PV had both similar and distinct protein and gene expression patterns that are related to their inherent physiological properties. These findings are important for us to better understand the physiological microenvironments of PV leaflet and valve cells for correctively engineering age-specific heart valve tissues.

## Introduction

Heart valves are complex soft tissues that control unidirectional blood flow within the heart^1^. There are four heart valves in the human heart, i.e. mitral valve (MV) and tricuspid valve (TV), which are atrioventricular valves, and aortic valve (AV) and pulmonary valve (PV), which are semilunar valves. Heart valve disease (HVD) is a common manifestation of cardiovascular disease and is a major cause of cardiovascular morbidity and mortality worldwide^2,3^. HVD affects more than 1 in 50 people and accounts for approximately 25,000 deaths annually in the US alone^4^. HVD can develop before birth (congenital) or can be acquired during a person's lifetime. Congenital HVD affects a significant portion of newborns, attributing to more than 48,000 cases annually^5^. The prevalence and severity of acquired HVD are highly related to age. The prevalence was less than 2% before the age of 65 years and then remarkably increased to 13.2% after the age of 75 years^6^. The physiochemical properties, extracellular matrix (ECM) components and properties, and cellular phenotypes and functions of heart valves dynamically change with maturation and aging^7–9^.

Among the four heart valves, the AV is the most prone to disease and thus is the most intensely studied cardiac valve with increasing the age. For example, Dr. Grande-Allen’s group systematically compared the AV proteoglycans (PGs) and glycosaminoglycans (GAGs) with age and demonstrated that many PG and GAG compositions and distributions were changed with maturation and aging^10^. The same group also demonstrated that aging elevated stiffness for radial and circumferential AV strips and stress relaxation in circumferential AV strips^8^. In contrast to the AV, very little is known about the age related structural and mechanical changes that occur in the PV^11,12^. The PV is of primary concern because of its involvement in common congenital heart defects. The PV itself is dysfunctional from birth in a number of common congenital conditions, including tetralogy of fallot and truncus arteriosus^13^. In addition, the PV is usually the site for prosthetic replacement following a Ross operation (a diseased AV is replaced with the patient’s own PV). The pulmonary autograft, or Ross operation, is used to replace the diseased aortic valve, and the pulmonary valve is replaced with a prosthetic valve^14,15^. Many tissue-engineered heart valves are targeting at repairing or regenerating the pulmonary heart valve in order to be instead of using prosthetic valves (either mechanical or biological) in the Ross operation. Therefore, it is also important and necessary to study the effects of age on the properties of PV to better understand the pathophysiology of PV disease and the target for tissue regeneration.

Both the PV and AV are composed of three semilunar cusps (or “leaflets”), which are mainly populated with valve interstitial cells (VICs). It is well known that VICs exhibit a heterogeneous but largely quiescent fibroblastic phenotype in healthy adult valve leaflets^16,17^. However, VICs can become activated and differentiate into a myofibroblastic phenotype in response to injury and microenvironmental cues^18,19^ and thus regulate the pathobiological responses of the valves^20,21^. Although many studies have extensively investigated the effects of microenvironments on VIC phenotypes and the interactions between VICs and ECM^22–24^, it is largely unknown about the effects of age on intrinsic VIC phenotypes.

In this study, we isolated PV leaflets from porcine hearts in different age groups (~ 4–6 months vs. ~ 2 years) and studied the effects of age on ECM components and mechanical properties of isolated PV leaflets. We also conducted proteomic analysis to screen the alteration of protein expressions in PV leaflets of both age groups. We further isolated primary pulmonary VICs (PVICs) from pig and performed RNA sequencing to analyze the global changes in gene expressions.

## Results

### Histological structures and ECM components

**Figure 1:**
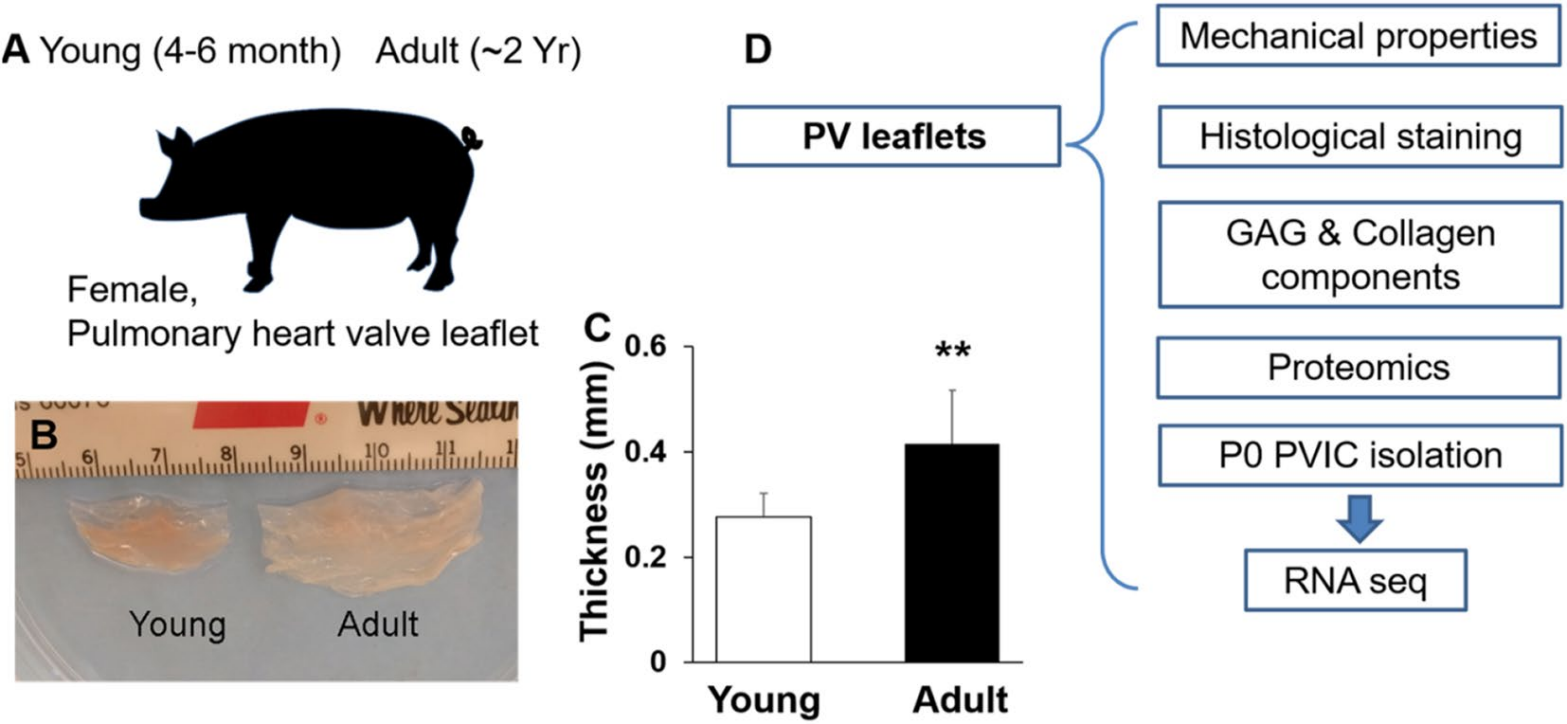
Isolation of porcine PV leaflets and experimental design. (**A**) PV leaflets were isolated from female pigs with ages of 4–6 months and 2 years (denoted as young and adult, respectively). (**B**) isolated PV leaflets from young and adult pigs; (**C**) thickness of young and adult PV leaflets (measured around free edge of valve cusp, 5 PV leaflets from three pigs for each age group were used, **p < 0.01); (**D**) schematic illustration of experimental design in this study. The isolated PV leaflets were subjected to mechanical properties testing, histological staining, ECM component measurement, proteomic analysis, and PVIC isolation for RNA seq.

PVs from 4–6-month and 2-year old pigs (denoted as young and adult, respectively) were isolated, and PV leaflets were further dissected (Fig. 1A). For domestic pig, an age of 4–6 months is equivalent to ~ 7–10 years for a human and 2-year-old porcine is equivalent to ~ 22 years for a human. The adult PV leaflets were much larger (Fig. 1B), as expected, and were also much thicker (Fig. 1C). The overall experimental designs were shown in Fig. 1D.

**Figure 2:**
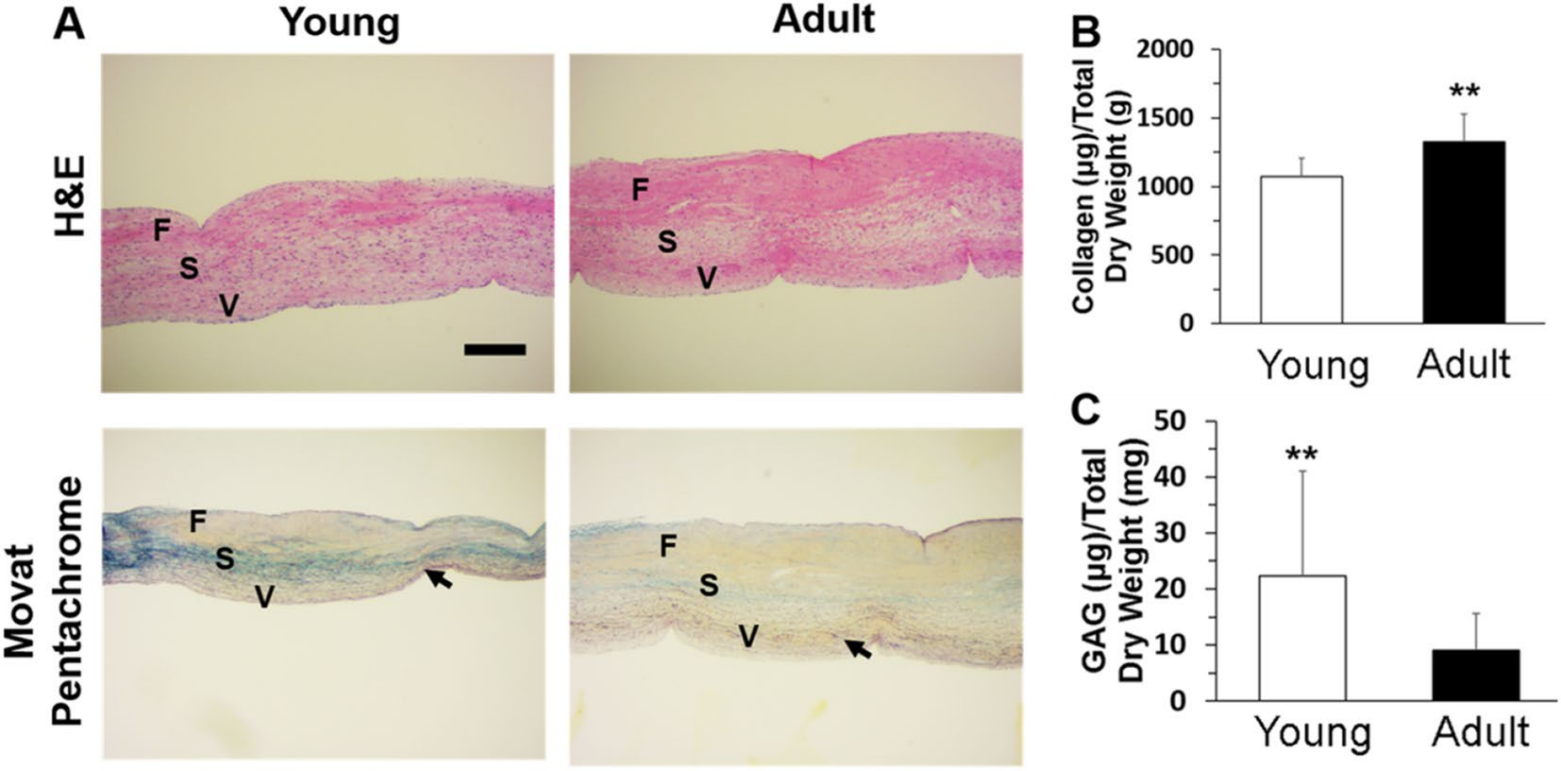
Histological staining and quantitative analysis of ECM components of young and adult PV leaflets. (**A**) H&E and Movat's pentachrome staining (*F* fibrosa, *S* spongiosa, *V* ventricularis; arrows indicate elastin fibers); Quantitative analysis of collagen (**B**) and GAGs (**C**) by using hydroxyproline and DMMB assays, respectively (five PV leaflets from 3 different pigs, two pieces from each PV leaflets, **p < 0.01).

We then conducted hematoxylin and eosin (H&E) and Movat's pentachrome staining to explore histological architectures of PV leaflets. The PV leaflets from adult pigs were much thicker compared to their young counterparts, as expected. Similarly to AV leaflets^25^, PV leaflets from both young and adult pigs showed internal heterogeneous structures with three layers, i.e. fibrosa (F), spongiosa (S), and ventricularis (V), as shown in Fig. 2A. Movat's pentachrome staining revealed that adult PV leaflets had thicker fibrosa layers with more collagen (yellow), while young PV leaflets had increased GAG components in the spongiosa layers (blue). Elastic fiber fragmentation with radially-aligned structure was observed within the ventricularis layer (black), with comparable thicknesses in both young and adult age groups. We further quantitatively evaluated the total collagen and GAG components in the PV leaflets. As shown in Fig. 2B,C, young PV leaflets had significantly less collagen and more GAGs compared to their adult counterparts, which is consistent with the histological staining results.

### Mechanical characterization of PV leaflets from young and adult pigs

**Figure 3:**
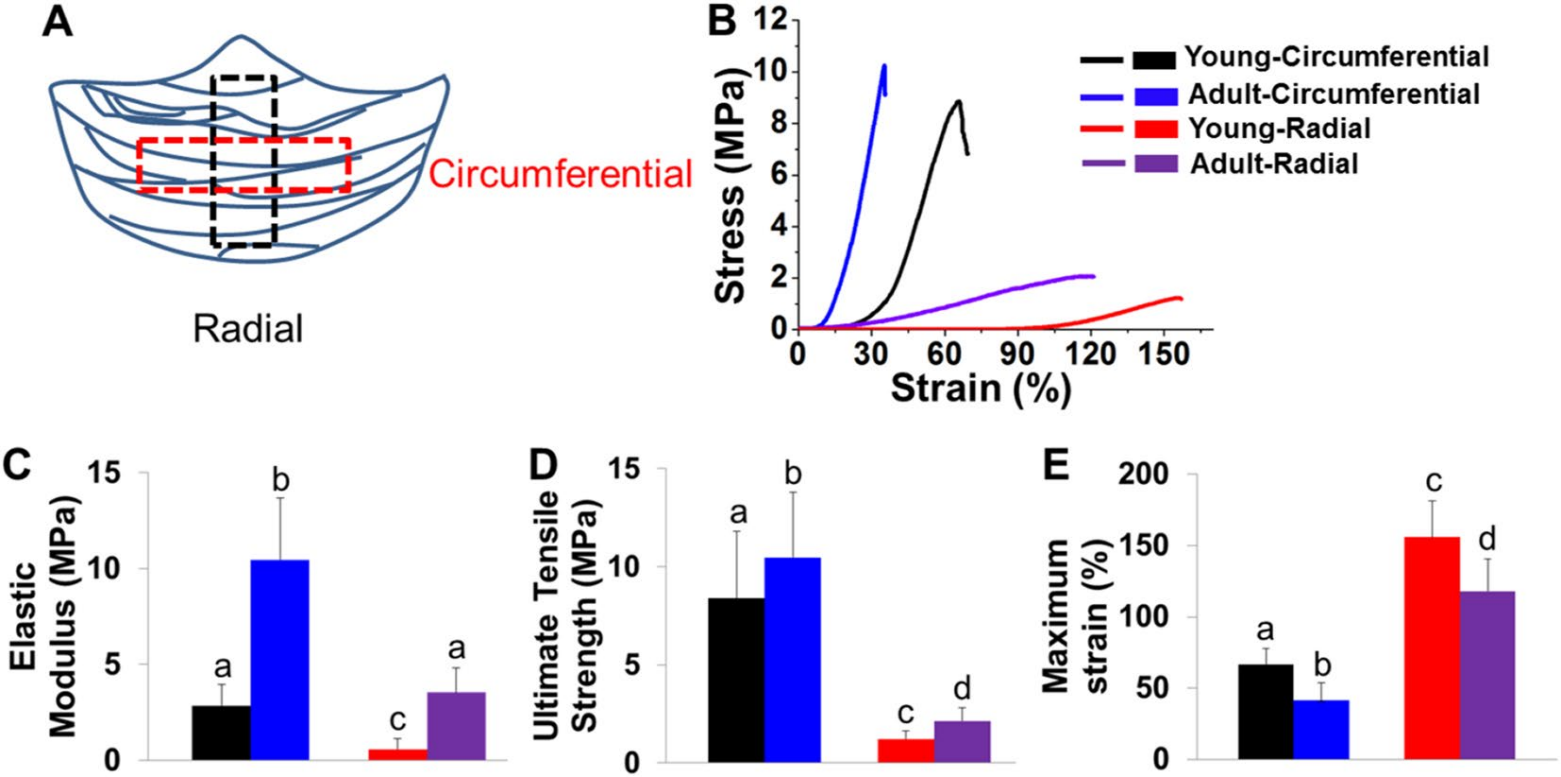
Uniaxial tensile mechanical properties of PV leaflets. (**A**) Preparation and cutting direction of PV leaflets. PV leaflets were cut out in the radial or circumferential direction (*F* fibrosa, *S* spongiosa, *V* ventricularis); (**B**) representative uniaxial tensile stress–strain curves for young and adult PV leaflets in circumferential and radial directions; summarized elastic modulus (**C**), ultimate stress (**D**), and ultimate strain (**E**) (5 PV leaflets from three pigs for each age group, bars that do not share letters are significantly different from each other).

We conducted uniaxial tensile tests to evaluate the mechanical properties of PV leaflets from young and adult pigs. The valve leaflets were cut into strips along radial and circumferential directions, as shown in Fig. 3A. The results of the tensile tests showed a nonlinear stress–strain behavior (Fig. 3B). For the circumferential direction, the tensile stress–strain curve had four regions, indicating four phases^26^, i.e. (1) low stress-low strain pretransition linear elastic phase, which is related to the straightening of the crimped fibers of collagen and elongating elastin fibers; (2) the highly non-linear transition phase, which is related to the force transfer from the elastin to the collagen fibers; (3) a post-transition linear elastic region related to elastic and collagen fiber elongation; (4) a non-linear region of decreasing stress due to elastin and collagen fibers rupture. However, for the radial direction, the phase 2 was not obviously observed, probably due to the lower elastin content in the radial direction. The PV leaflets from both young and adult pigs had higher elastic moduli and ultimate stresses and smaller ultimate strains in the circumferential direction than the radial direction (Fig. 3C–E). The adult PV leaflets had statistically higher elastic modulus and ultimate stress in both directions than young PV leaflets, while the young age group had a much larger ultimate strain. This is probably because of the increase of collagen in adult PV leaflets.

### Proteomics analysis of PV leaflets

**Figure 4:**
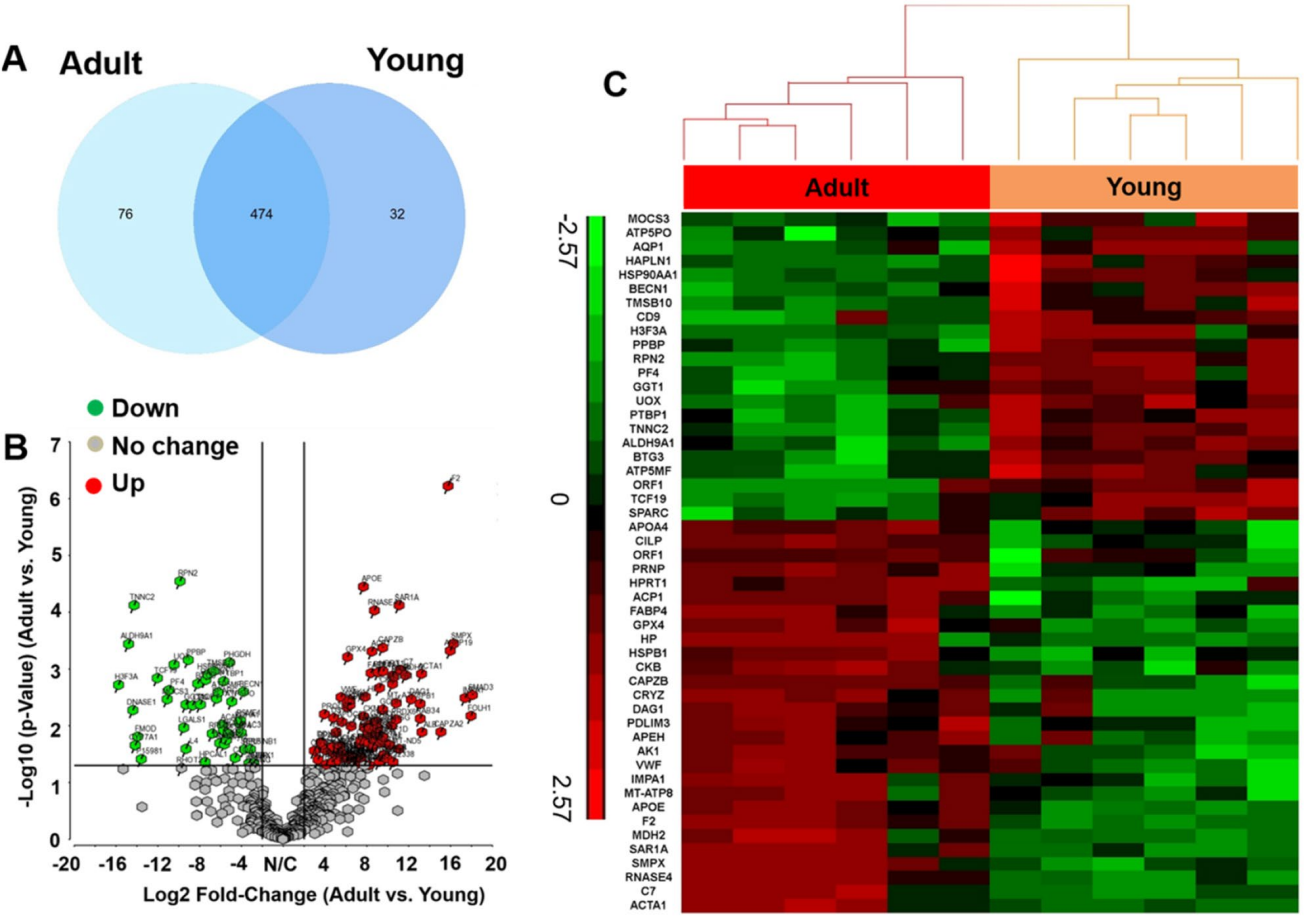
Proteomic profiles of PV leaflets from young and adult pigs. (**A**) The Venn diagram illustrates the overlap and unique proteins identified in young and adult groups; (**B**) volcano plot showing –log (p-value) versus protein fold change (Log2) of all identified proteins. (Red, 60 up-regulated proteins; green, 27 down-regulated proteins; grey, not significantly changed for Adult vs. Young). The solid lines represent a twofold difference in abundance and a p-value < 0.05. (**C**) Heatmap of the 50 most upregulated and downregulated proteins.

Proteomics analysis allowed an overall identification of 542 proteins, and of these proteins, 474 proteins were expressed in both age groups, while 32 and 76 proteins were unique to young and adult, respectively, as shown Fig. 4A. We further selected differentially expressed proteins based on fold change (FC ≥ 2), and the p-value < 0.05. Figure 4B shows a volcano plot representing the relation between the protein abundance, expressed as Log2 FC, and the p-value, expressed as –Log10 p-value. Among these proteins, 87 proteins were chosen, with 27 proteins upregulated (labeled in red) and 60 downregulated (labeled in green) in the young group, compared to the adult group. We further chose 50 of the most upregulated and downregulated proteins, based on label-free quantitation (LFQ) intensities, and generated a heatmap, as shown in Fig. 4C.

**Figure 5:**
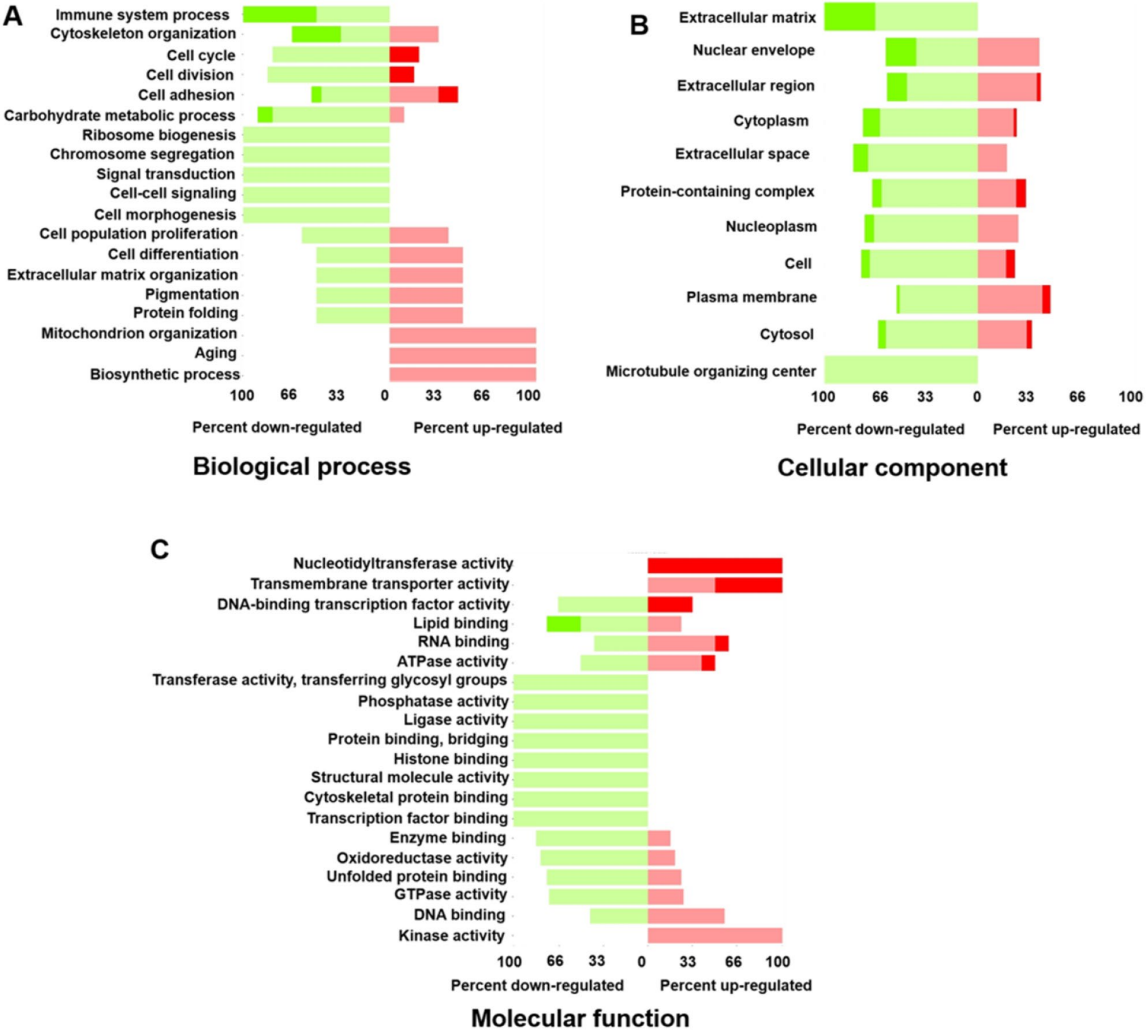
GO enrichment analysis of differentially expressed proteins in young and adult PV leaflets. GO molecular function (**A**), biological process (**B**), and cellular component (**C**) enrichment analyses of up-regulated and down-regulated proteins (Adult vs. Young, dark color represents proportions that passes FDR of 0.05, indicating statistical significance).

To identify the key pathways altered with maturation and age, we performed Gene Ontology (GO) analysis, as shown in Fig. 5. GO analysis suggested that the young group expressed more proteins related to transferase activity, phosphatase activity, ligase activity, structural molecule activity, protein binding, histone binding, and transcription factor binding in molecular function, while the adult group had more proteins related to nucleotidyl transferase activity, transmembrane transporter activity, and kinase activity (Fig. 5A). In addition, the proteins related to immune system process, ribosome biogenesis, chromosome segregation, signal transduction, cell–cell signaling, and cell morphogenesis in the biological process were upregulated in the young group, and proteins related to mitochondrion organization, aging, and biosynthetic process were upregulated in the adult group (Fig. 5B). Many proteins in the category of cellular component, like extracellular matrix and microtubule organizing center, were downregulated in the adult group compared to young group (Fig. 5C).

### Functional characterization of differentially expressed genes (DEGs) in RNA sequencing analysis

**Figure 6:**
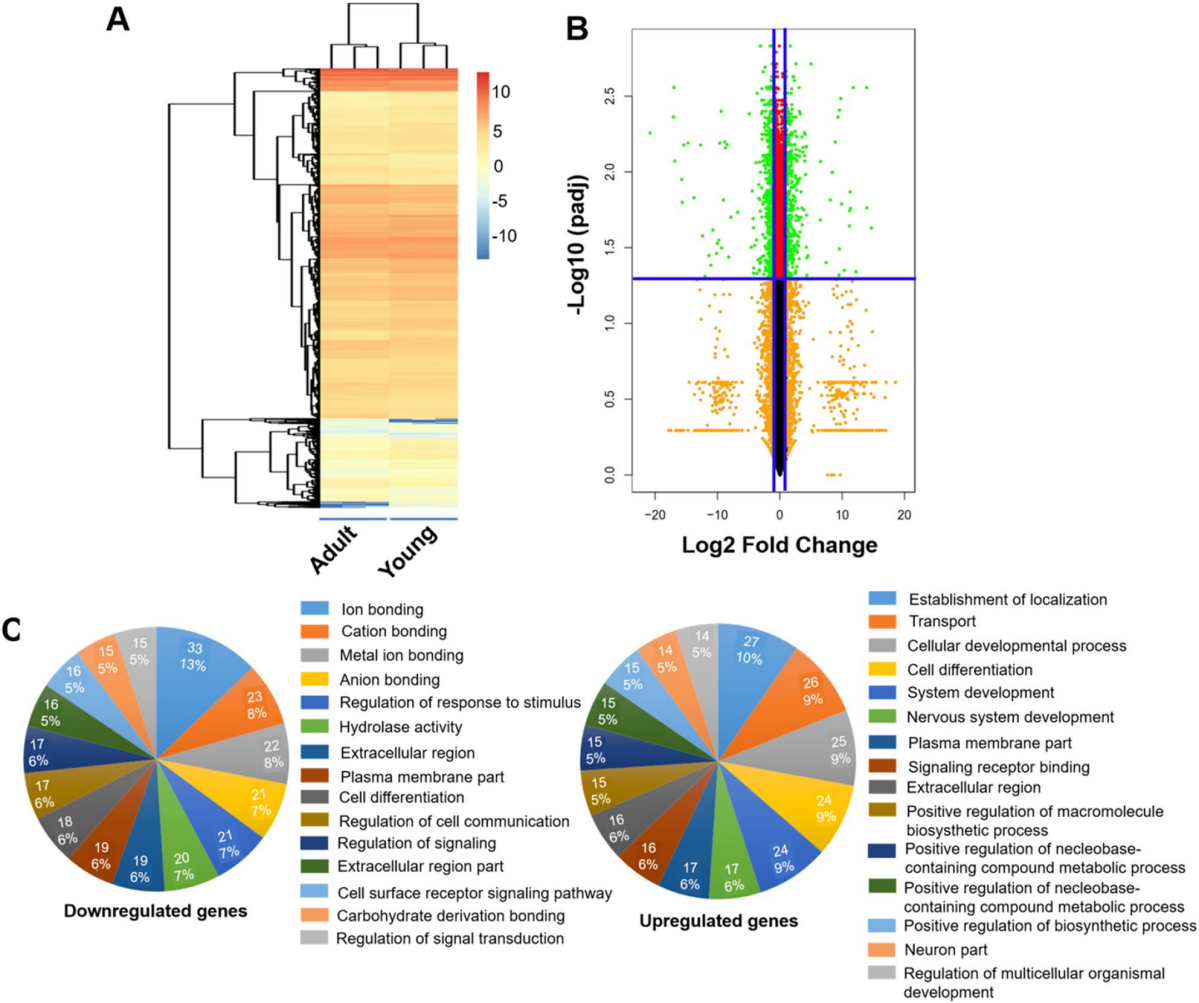
RNA sequencing reveals global gene expression alterations for P0 PVICs from young and adult PV leaflets. (**A**) Overall heatmap; (**B**) Volcano plot (Adult vs. Young); (**C**) Pie chart representation of GO analysis (Adult vs. Young).

We further isolated and cultured P0 PVICs from PV leaflets in young and adult pigs for global RNA sequencing. To visualize overall transcriptomic differences between these two age groups, an overall heatmap was generated, including all of the identified significant genes (adjusted *p* ≤ 0.05) (3835) (Fig. 6A). Hierarchical clustering showed that PVICs from young and adult PV leaflets were well distinguished with all three of the subjects correctly classified. A volcano plot was also used to evaluate gene expression variation between the young and adult age groups (Fig. 6B). Genes with adjusted *p* ≤ 0.05 and log2 FC ≥ 2 or ≤ − 2 were identified as significantly differently expressed. The top 20 upregulated and downregulated genes were sorted by p-value and the heatmap was presented (Fig. S1). To further correlate the relevance of these genes, pie charts of the GO analysis were generated to depict the upregulated and downregulated gene categories for adult group versus young group, as shown in Fig. 6C. Significantly enriched GO categories in the young age group included ion binding, regulation of response of stimulus, hydrolase activity, etc., while the GO categories that were upregulated in the adult age group included the establishment of localization, transport, cellular development process, cell differentiation, etc.

**Figure 7:**
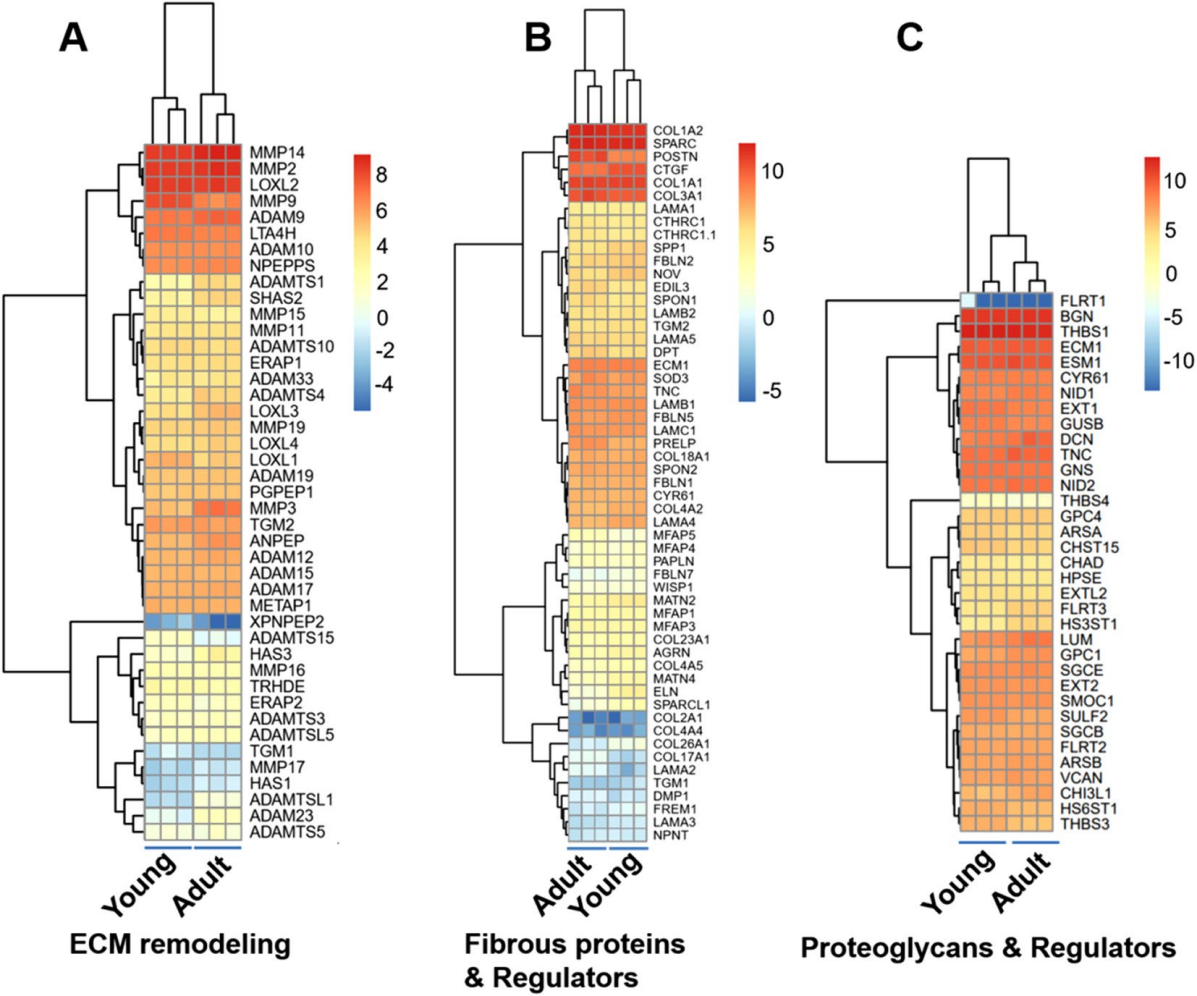
Heatmaps of selected gene expressions related to ECM remodeling (**A**), fibrous proteins and regulators (**B**), and proteoglycans and regulators (**C**).

VICs have close interactions with their ECMs, and the VIC-ECM interplay significantly regulates VIC phenotypes^27,28^. Therefore, we focused on several ECM related categories to better understand the major differences between PVICs isolated from young and adult pig PV leaflets. As shown in Fig. 7, heat maps were specifically generated to compare gene expressions involved in ECM remodeling, fibrous proteins and regulators, and proteoglycans and glycoproteins. In this analysis, many genes related to ECM remodeling (Fig. 7A) were significantly upregulated by the PVICs from adult groups, including a disintegrin and metalloprotease domain (ADAM)9, 12, 23, a disintegrin and metalloproteinase with thrombospondin motifs (ADAMTS)1, 4, hyaluronan synthase (HAS)2, 3, matrix metallopeptidases (MMP)3, 19, alanyl aminopeptidase, membrane (ANPEP), and lysyl oxidase like (LOXL)3, 4. In contrast, PVICs from the young group only significantly upregulated the expression of MMP9, LOXL1, and ADAMTS15. Fibrous proteins are proteins with long and narrow strands and provide structural support for cells and tissues. These include collagen (COL), elastin (ELN), laminin (LAM), microfibril associated protein (MFAP), fibulin (FBLN), et al. There are many regulators, like growth factors and cytokines, which can regulate the fibrous protein formation, deposition, binding, and organization. We found that PVICs from the young age group significantly upregulated the expressions of ELN, FBLN2, COL26A1, connective tissue growth factor (CTGF), secreted phosphoprotein (SPP)1, nephroblastoma overexpressed (NOV), and SPARC-like protein (SPARCL)1 (Fig. 7B). The PVICs from the adult age group upregulated the expressions of COL17A1, LAMA1, proline and arginine rich end leucine rich repeat protein (PRELP), EGF like repeats and discoidin domain (EDIL) 3, spondin (SOPN)1, periostin (POSTN), and dentin matrix acidic phosphoprotein (DMP)1. Apart from fibrous proteins in ECM, proteoglycans (PGs) are also important ECM components in the valve leaflets. PGs can interact with other ECM structural proteins, such as collagen and elastin, and such bindings and interactions profoundly affect valve biomechanical and molecular processes. As shown in Fig. 7C, PVICs from the adult group increased the expressions of fibronectin leucine rich transmembrane protein (FLRT)3, heparan sulfate-glucosamine 3-sulfotransferase 1 (HS3ST1), Decorin (DCN), Tenascin C (TNC), and chitinase 3 like 1 (CHI3L1), while young PVICs upregulated thrombospondin (THBS)3 and HS6ST1. PVICs from both age groups highly expressed MMP2, 14, LOXL2, COL1A1, COL1A2, biglycan (BGN), and thrombospondin (THBS)2. The differences in gene expressions were also analyzed by Ingenuity Pathway Analysis (IPA). Fig. S2 and Table S1 show the top related canonical signaling pathways, and the top upstream regulators are TGFB1, TNF, dexamethasone, beta-estradiaol, and TP53 (Table S2).

## Discussion

It is well-known that maturation and aging significantly affect valve structure and mechanical properties, ECM components and remodeling, and valvular cell phenotypes^29–31^. These alterations together are major contributors to HVD. Although some comprehensive studies related to changes in tensile properties of the adult porcine mitral and aortic valves^9,32^ and ECM distribution^10^ have already been reported, the systematical studies of effects of age on PV properties in large animals and humans are still limited. In this study, we isolated PV leaflets and P0 PVICs from young and adult pigs and compared their properties. There are several major reasons for us to choose these two age groups. First, PV diseases are most often found in infants as the congenital diseases that developed during the fetal stage. A child with less severe PV disease will have the repair scheduled, while some children may need to have a PV replacement. The children with less severe PV diseases may also need a PV replacement as a teen or young adult to prevent complications. This is why we need better understand the properties and biological features of PV in the young age group. Second, for many children and young adults with diseased AV or AV failure, a Ross operation (a diseased AV is replaced with the patient’s own PV) is usually performed, and the PV is the site for further prosthetic replacement. In addition, the clinical need for a living valve replacement is greatest for pediatric populations, where growth and biological integration is essential. A tissue engineered heart valve (TEHV) thus gains more and more attention. However, most studies use adult valves as the TEHV target. Many studies generated TEHV with the mechanical properties, material components, and other biophysiochemical properties similar to adult valves, rather than mimicking young valves. This may cause many problems after implantation, resulting in calcification and failure. Therefore, the information and knowledge related to young PV will provide correct target to design the prosthetic PV replacement and tissue engineered valves. In order to ensure long-term functionality of TEHVs, identifying the differences in the psychological aspects between young and adult age groups is of the greatest importance.

Compared to AV leaflets, PV leaflets were reported to be less stiff and more extensible in the radial direction, probably because AV leaflets have a higher collagen content and are required to sustain higher pressures^33^. Similarly, to AV leaflets, we found that the size and thickness of PV leaflets increased with increasing age from young to adult. We also demonstrated that the modulus and stress increased, while the strain decreased in both the radial and circumferential directions from young to adult age groups. These results are consistent with the reports that studied the effects of age on AV mechanical properties^9,34^. The alterations of the mechanical properties are highly correlated with the ECM component and distribution changes. We found that adult PV leaflets had more collagen and less GAGs compared to the young group. Stephens et al. also demonstrated that the collagen in the porcine AV and MV increased from the 6-week to 6-month age groups^8^. Bashey et al. reported that the collagen concentrations in human AV, MV, and TV showed a decreasing trend from age group of 20–30 to age group of 80^35^. Therefore, it is possible that the collagen content increases from young to adult and then gradually decreases with aging. Stephens et al. also demonstrated that young pig AV leaflets had a higher concentration of GAGs, and the general aging trends included an increase in hyaluronan and in iduronate-containing GAGs^10^.

We further conducted proteomics and RNA sequencing to discover the alteration of global expression profiling in protein and gene levels. We found that both young and adult groups had inherent expression patterns. For example, adult PV leaflets and PVICs expressed much more osteogenesis related proteins/genes (like cartilage intermediate layer protein (CILP), POSTN, and DMP1) and ECM remodeling proteins/genes. This indicates that adult PV leaflets may be intended to undergo pathological changes. On the other hand, young PV leaflets and PVICs upregulated many physiological protective proteins/genes, like heat shock protein 90 alpha family class A member 1 (HSP90AA1, related to cellular response and recovery), SPP1 (related to bone resorption), and beclin 1 (BECN1, related to autophagy), which have been demonstrated to be beneficial for directly protecting valve cells or slowing down the pathological progression through a compensatory mechanism^36–39^. Balaoing et al. evaluated hemostatic protein regulation in AV tissues and porcine valve endothelial cells (PVECs) with age and reported that old AV leaflets expressed more von Willebrand factor (vWF), while PVECs from the young age group had more gene expression of vWF^40^. Our study also demonstrated that adult PV leaflets upregulated the vWF and thrombin/prothrombin expressions, and young PV leaflets had higher expressions of pro-platelet basic protein (PPBP) and platelet factor 4 (PF4). These results also indicate that hemostatic proteins change with age. Young PVICs significantly upregulated ELN and related genes (like FBLN2 and LOXL1), which may explain why young PV leaflets are more stretchable. Wyss et al. demonstrated that the VIC elastic modulus increased with culture passage numbers on stiff tissue culture substrates^41^. Therefore, we only implemented P0 PVICs for the RNA seq. However, Wang et al. also demonstrated that P0 VICs cultured on stiff cell culture plates alter the mRNA expression genome-wide, compared with freshly isolated VICs^42^. Although we also tried to isolate total RNA from fresh PV leaflets, we failed to obtain enough samples with high quality for the RNA seq.

Our results in the current study can be taken into account in the design of TEHVs, for example, by mimicking the biomechanical properties and adapting cellular and ECM microenvironments. Our current study provided more insights into how valvular properties and protein/gene expressions alter from young to adult ages and provided targets to engineer age-specific tissues. Many studies have suggested that ECM remodeling is mediated by the VICs, which, in return, further regulate VIC phenotypes^43–46^. An appropriate recapitulation of the ECM environment will direct the valvular cells into functional regeneration of heart valve tissue, while simultaneously preventing pathological alteration. One of the limitations of this this study is that the PV leaflets from the old age group were not included due to the lack of availability of tissues. Another limitation is that only female pigs were used due to the availability. Future studies may consider other age groups and sex group and include the valves from pathological diseases conditions.

## Conclusions

In this study, we isolated PV leaflets from young and adult age pigs and systematically compared their thickness, mechanical properties, and ECM components. We also conducted proteomics and RNA seq to investigate the global changes in protein and gene levels of PV leaflets and P0 PVICs. Our data suggests that the size and thickness of PV leaflets increased from young to adult. The elastic modulus and ultimate stress in both the radial and circumferential directions also increased, whereas the ultimate strain decreased when age increased. Young PV leaflets had more GAGs, while their adult counterparts had more collagen. Both young and adult PVs had both similar and distinct protein and gene expression patterns. Adult PV leaflets and PVICs expressed much more osteogenesis related and ECM remodeling proteins/genes, while young PV leaflets and PVICs upregulated many physiological protective proteins/genes. Overall, this study generates insights into how age alters PV leaflet and PVIC properties and provides a template to mimic ECM and the cellular microenvironment for engineering age-specific heart valve tissues.

## Materials and methods

### Isolation of porcine PV leaflets

Fresh female porcine hearts were obtained from Tissue Source LLC and delivered overnight. PVs from 4–6-month and 2-year old pigs (denoted as young and adult, respectively) were isolated, and the PV leaflets were further dissected. The PV leaflets were subjected to mechanical properties testing, histological staining, ECM component measurement, proteomic analysis, and VIC isolation, as shown in Fig. 1. For the thickness measurement, since the PV leaflets have a heterogenous structure, we measured the thickness of the leaflet edge of valve cusp using electronic calipers.

### Histological staining

PV leaflets were fixed in buffered formalin for 24 h at 4 °C, followed by routine dehydration and paraffin embedding. Slides with 5 μm thickness were sectioned, deparaffinized, and stained with H&E and Movat's pentachrome to depict matrix architecture.

### ECM components analysis

Dimethylmethylene blue (DMMB) assay was performed to measure the sulfated GAGs in the PV leaflets of different age groups^47^. The tissue samples (five PV leaflets from different pigs, two pieces from each PV leaflet) were digested in the 50 mM phosphate buffer containing 300 µg/ml papain, 5 mM cysteine, and 5 mM EDTA for 16 h at 60 °C. GAG concentration was calculated by calibrating against a standard curve obtained with shark chondroitin sulfate (Sigma). The total collagen content was determined using the hydroxyproline assay^48^. Briefly, the tissue samples were hydrolyzed by HCl, and then the dried hydrolyzates were then treated with chloramine T reagent for oxidation. The Ehrlich’s aldehyde reagent was reacted with the hydrolyzed samples at 65 °C for 20 min in order to generate chromospheres. The amount of hydroxyproline was measured with a microplate reader at 550 nm (n = 5). The contents of GAG and collagen were normalized using the dry weight of the tissue samples and expressed in units of µg/total dry weight mg.

### Mechanical characterization of PV leaflets

PV leaflets from different age groups were cut into rectangular shapes (15 mm × 2.5 mm) along with circumferential and radial directions, as shown in Fig. 2A. The thickness of the tissue was measured at three different locations. The mechanical properties of PV leaflet strips cut in circumferential and radial directions were examined at room temperature by a tensile strength tester (CellScale, Canada) with a gauge length of 5 mm and a constant movement rate of 10 mm/min until failure occurred. We calculated the elastic stiffness of the scaffolds from the initial 5–10% strain region of the stress–stain curves. The ultimate tensile strength and maximum strain were also determined based on the stress–stain curves.

### Protein digestion, liquid chromatography (LC)-tandem mass spectrometric (MS/MS), and data analysis

The PV leaflet samples from three biological replicates per group were cut into small pieces and digested in lysis buffer. Protein digestion process was followed previous reported method with certain modification^49^. The detergent was removed by chloroform/methanol extraction, and the protein pellet was re-suspended in 100 mM ammonium bicarbonate and digested with MS-grade trypsin (Pierce) overnight at 37 °C. Peptides cleaned with PepClean C18 spin columns (Thermo Scientific) were re-suspended in 2% acetonitrile (ACN) and 0.1% formic acid (FA), and 500 ng of each sample were loaded onto trap column Acclaim PepMap 100 75 µm × 2 cm C18 LC Columns (Thermo Scientific) at a flow rate of 4 µl/min. Then they were separated with a Thermo RSLC Ultimate 3000 (Thermo Scientific) on a Thermo Easy-Spray PepMap RSLC C18 75 µm × 50 cm C-18 2 µm column (Thermo Scientific) with a step gradient of 4–25% solvent B (0.1% FA in 80% ACN) from 10–100 min and 25–45% solvent B for 100–130 min at 300 nL/min and 50 °C with a 155 min total run time. Eluted peptides were analyzed by a Thermo Orbitrap Fusion Lumos Tribrid (Thermo Scientific) mass spectrometer in a data dependent acquisition mode. A survey full scan MS (from m/z 350–1800) was acquired in the Orbitrap with a resolution of 120,000. The AGC target for MS1 was set as 4 × 10^5^, and the ion filling time set as 100 ms. The most intense ions, with charge states of 2–6, were isolated in a 3 s cycle and fragmented using HCD fragmentation with 35% normalized collision energy and detected at a mass resolution of 30,000 at 200 m/z. The AGC target for MS/MS was set as 5 × 10^4^, and the ion filling time was set at 60 ms. The dynamic exclusion was set for 30 s with a 10 ppm mass window. Each sample was run in duplicates. Protein identification was performed by searching MS/MS data against the swiss-prot Sus scrofa protein database downloaded on June, 10 2020 (1434 entries) using the in house PEAKS X + DB search engine. The search was set up for full tryptic peptides with a maximum of two missed cleavage sites. Acetylation of protein N-terminus and oxidized methionine were included as variable modifications, and carbamidomethylation of cysteine was set as a fixed modification. The precursor mass tolerance threshold was set at 10 ppm, and the maximum fragment mass error was 0.02 Da. The significance threshold of the ion score was calculated based on a false discovery rate of ≤ 1%. Quantitative data analysis was performed using progenesis QI proteomics 4.2 (Nonlinear Dynamics). Statistical analysis was performed using ANOVA, and The Benjamini-Hochberg (BH) method^50^ was used to adjust *p* values for multiple-testing caused false discovery rate. The adjusted *p* ≤ 0.05 was considered as significant. Partek Genomics Suite 7 was used to generate the heatmap and volcano plot.

### Isolation and culture of PVICs

PVICs were isolated from porcine PV leaflets, as described previously^23,51^. Briefly, the surfaces of the PV leaflets were digested with collagenase for 10 min to remove the endothelium. The remaining portions of the leaflets were then cut into small pieces (~ 1 mm × 1 mm) and digested with fresh collagenase solution for 2 h. We utilized five PV leaflets from 3 different pigs for each age group. After filtration and centrifugation, the cells were pooled together for each age group and seeded in three wells of 6-well plate to generate 3 replicates. In each group, the PVICs were cultured in Dulbecco’s Modified Eagle’s Medium (DMEM, Invitrogen) supplemented with 10% fetal bovine serum (FBS, Gibco) and 1% penicillin/streptomycin (PS, Gibco) at 37 °C and 5% CO_2_. Only P0 PVICs were used for further RNA sequencing.

### RNA sequencing

Total RNA was extracted from P0 PVICs isolated from young and adult porcine PV leaflets using RNeasy mini-kits (QIAGEN), according to the manufacturer’s instructions. All of the RNA samples had a 260/280 ratio > 2.0 and RNA Integrity Number > 9.0 verified by RNA Bioanalyzer. Libraries for RNA sequencing were prepared using the TruSeq RNA Sample Prep Kit (Illumina), and sequencing was performed on Sciclone G3 NGS Workstation.

### RNA seq data analysis

The original FASTQ format reads were merged and trimmed by the fqtrim tool (https://ccb.jhu.edu/software/fqtrim) to remove adapters, terminal unknown bases (Ns), and low quality 3′ regions (Phred score < 30). The trimmed FASTQ files were processed by FastQC (Andrews S. (2010). FastQC: a quality control tool for high throughput sequence data; available online at http://www.bioinformatics.babraham.ac.uk/projects/fastqc) for quality control. The trimmed FASTQ files were mapped by STAR^52^, as the aligner, and RSEM^53^, as the tool for annotation and quantification, at both gene and isoform levels. The normalized expression values in TPM (Transcripts Per Kilobase Million) at gene levels were subject to a student’s t-test for statistical comparisons. The Benjamini-Hochberg (BH) method^50^ was also used to adjust *p* values for multiple-testing caused false discovery rate. The adjusted *p* ≤ 0.05 was considered as significant.

### Hierarchical clustering analysis

We plotted hierarchical clustering heatmaps using the R package, pheatmap (version 1.0.12). The heatmaps were plotted based on the value of Log2 (TMP + 0.0001) for each gene in each sample to avoid any arithmetic error of Log2 (0).

### GO analysis

For the protein list from the proteomic data analysis, we used WebGestalt^54^ for the GO analysis in three categories: biological process, cellular component, and molecular function. For RNAseq comparison data, with the criteria of adjusted *p* ≤ 0.05 and log2 Fold Change (FC) ≥ 2 or ≤ -2 to obtain the up- and down-regulated gene lists, respectively, we performed GO analyses and pie chart plotting (each comparison results include 2 gene lists, up- and down-regulated). Each gene list was subject to ShinyGO v0.60: Gene Ontology Enrichment Analysis^55^ for in-depth analysis, which used all gene IDs and were mapped with the database Ensembl to find the Gene Ontology Enrichment Analysis, such as biological process, cellular components and molecular functions.

### Functional pathway analysis

The differentially expressed genes from RNAseq analysis were further analyzed using Ingenuity Pathway Analysis (IPA; QIAGEN Inc., https://www.qiagenbioinformatics.com/products/ingenuity-pathway-analysis). We performed canonical pathway analysis based on the identified the pathways that were most significant to the gene list from the IPA library. Two methods were used to measure the significance of the association between the gene list and the canonical pathway^56^: (1) a ratio of the number of genes from the list that map to the pathway divided by the total number of genes that map to the canonical pathway is displayed; and (2) a Fisher’s exact test was used to calculate a *p* value, determining the probability that the association between the genes in the list and the canonical pathway is explained by chance alone.

### Statistical analysis

All quantitative data are expressed as the mean ± standard deviation (SD). Statistical analysis was performed using ANOVA with Scheffé post-hoc tests. A value of *p* < 0.05 is considered to be statistically significant.

### Ethical approval

All experimental protocols were approved by University of Nebraska Medical Center. All the methods were carried out in accordance with the relevant guidelines and regulations.

## Supplementary Information

Supplementary Information 1.

Supplementary Information 2.

Supplementary Information 3.

Supplementary Information 4.

Supplementary Information 5.
