# Supplementary material for: Age related extracellular matrix and interstitial cell phenotype in pulmonary valves
Source: Sci Rep. 2020 Dec 7;10:21338. doi: 10.1038/s41598-020-78507-8 (PMC7721746; doi:10.1038/s41598-020-78507-8)
Supplement: Supplementary file 1 — Supplementary Information 1. [file 41598_2020_78507_MOESM1_ESM.doc]

**Supporting Information**

**Age related extracellular matrix and interstitial cell phenotype in pulmonary valves**

Shaohua Wu,^1,2^ Vikas Kumar,^3,4^ Peng Xiao,^4^ Mitchell Kuss,^2^ Jung Yul Lim,^5^ Chittibabu Guda,^4^ Jonathan Butcher,^6^ Bin Duan,^2,5,7,*^

^1^College of Textiles & Clothing, Qingdao University, Qingdao, People’s Republic of China

^2^Mary & Dick Holland Regenerative Medicine Program and Division of Cardiology, Department of Internal Medicine, University of Nebraska Medical Center, Omaha, NE, USA

^3^Mass Spectrometry and Proteomics Core Facility, University of Nebraska Medical Center, Omaha, NE, USA

^4^Department of Genetics, Cell Biology & Anatomy, College of Medicine, University of Nebraska Medical Center, Omaha, NE, USA

^5^Department of Mechanical and Materials Engineering, University of Nebraska-Lincoln, Lincoln, NE, USA

^6^Meinig School of Biomedical Engineering, Cornell University, Ithaca, NY, USA

^7^Department of Surgery, College of Medicine, University of Nebraska Medical Center, Omaha, NE, USA

*Corresponding author

E-mail address: [bin.duan@unmc.edu](mailto:bin.duan@unmc.edu)

Phone: (402) 559-9637

**This file includes:**

Supplemental Figure (2)

Supplemental Table (2)

Supplemental Figure 1

Heatmap of the 20 most upregulated and downregulated genes in PVICs isolated from young and adult PVs.

Supplemental Figure 2

Top canonical pathways enriched by Ingenuity Pathway Analysis (IPA). The orange vertical line running through the bars is threshold for P value. Bars indicate significance levels of individual pathways.

Supplemental Table 1

Summarized top canonical pathways

Supplemental Table 2

Identified top upstream regulators by IPA
