# Supplementary figures and images for: Age related extracellular matrix and interstitial cell phenotype in pulmonary valves
Source: Sci Rep. 2020 Dec 7;10:21338. doi: 10.1038/s41598-020-78507-8 (PMC7721746; doi:10.1038/s41598-020-78507-8)

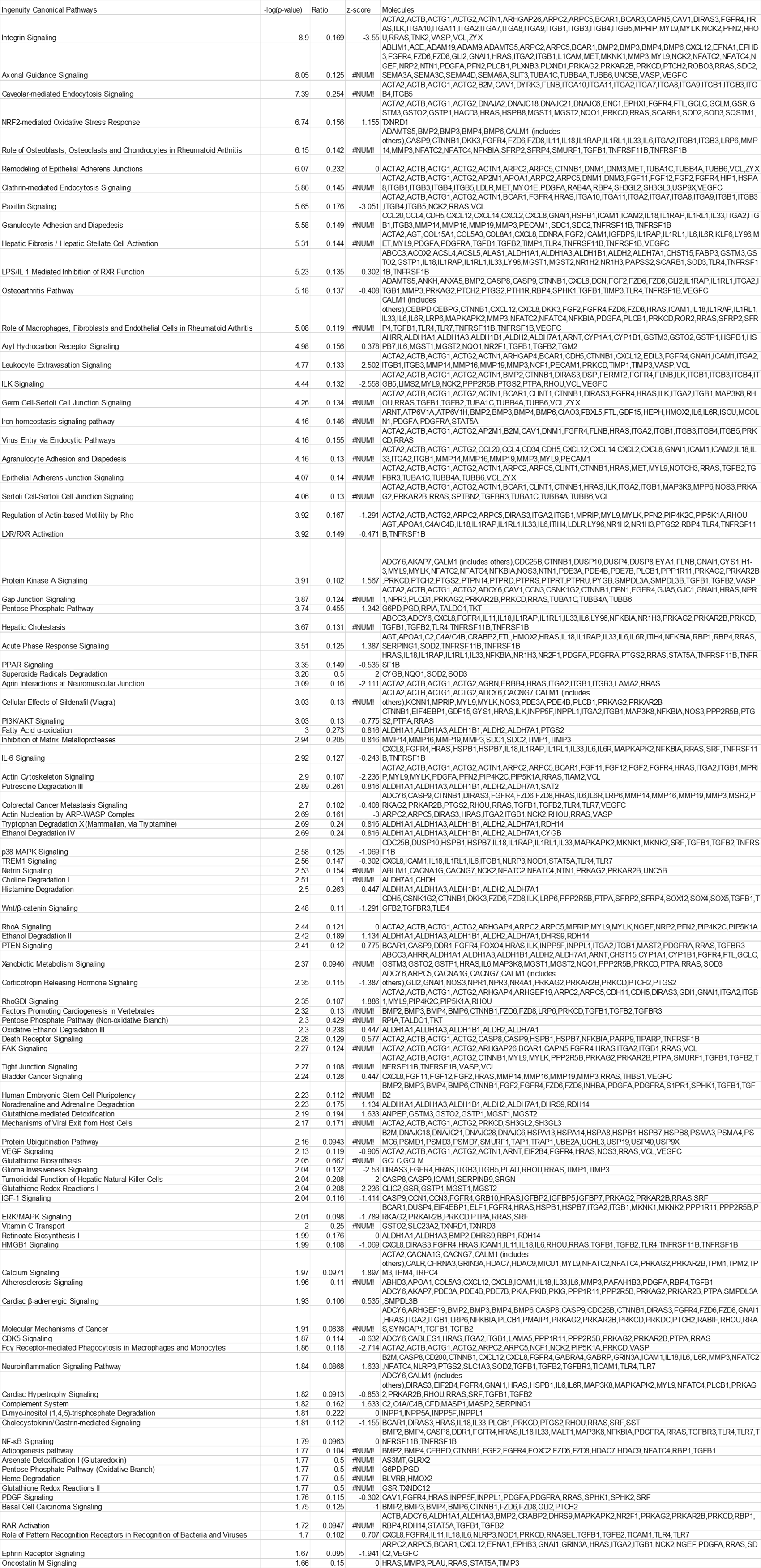

Supplement: Supplementary file 2 — Supplementary Information 2. [file 41598_2020_78507_MOESM2_ESM.tif]

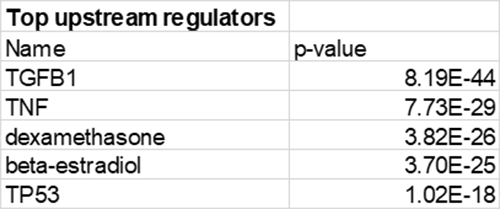

Supplement: Supplementary file 3 — Supplementary Information 3. [file 41598_2020_78507_MOESM3_ESM.tif]

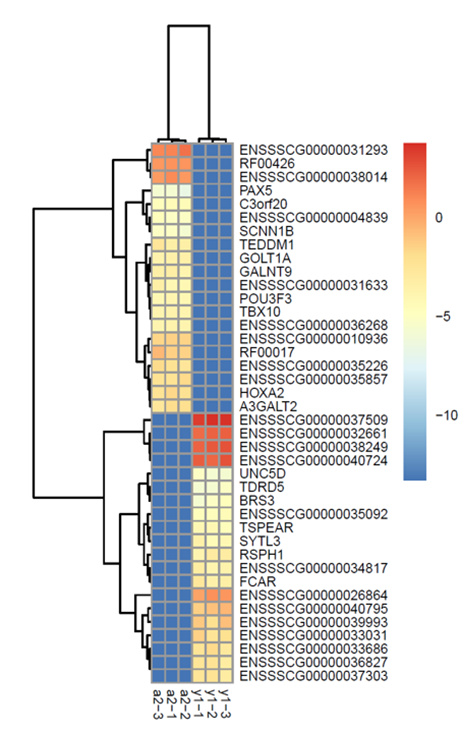

Supplement: Supplementary file 4 — Supplementary Information 4. [file 41598_2020_78507_MOESM4_ESM.tif]

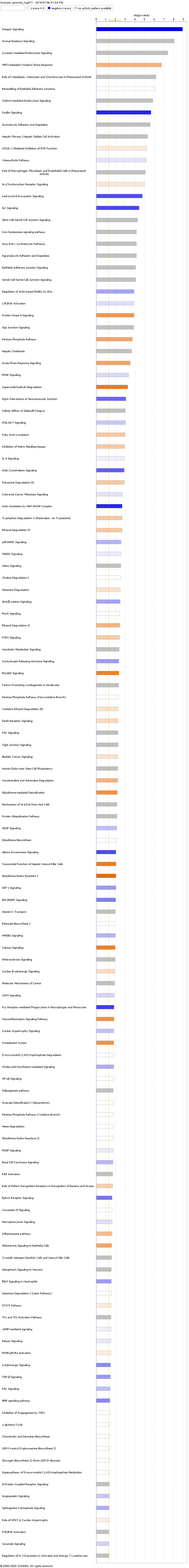

Supplement: Supplementary file 5 — Supplementary Information 5. [file 41598_2020_78507_MOESM5_ESM.tif]
